# The innovation of spinal endoscopy: one-hole bi-medium endoscopy for decompression and fusion in the treatment of lumbar degenerative diseases

**DOI:** 10.3389/fsurg.2025.1583156

**Published:** 2025-09-10

**Authors:** Xingang Cui, Hengren Li, Xingzhi Jing, Fei Jia, Xiaoyang Liu

**Affiliations:** ^1^ Department of Spine Surgery, Shandong Provincial Hospital, Shandong University, Jinan, China; ^2^ Department of Spine Surgery, Shandong Provincial Hospital Affiliated to Shandong First Medical University, Jinan, China

**Keywords:** endoscopy, fusion, decompression, lumbar degenerative diseases, minimally invasive surgery

## Abstract

The spinal endoscopy technique has been widely used in the treatment of lumbar, thoracic, and cervical diseases over the past 20 years. Minimally invasive decompression, assisted by fixation and interbody fusion, is the optimal treatment for lumbar degenerative diseases, such as lumbar spondylolisthesis and lumbar spinal stenosis. Although endoscopic lumbar spinal interbody fusions have been reported, further evaluation of their effectiveness and efficacy is necessary. We innovated the spinal endoscopy technique called the one-hole bi-medium endoscopy (OBE) technique, in which lumbar interbody fusion, pedicle screw fixation, and bilateral decompression are completed through one incision under endoscopic visualization with or without liquid irrigation. The visual analog scale and Oswestry disability index scores were significantly improved after the OBE treatment. Our study concludes that the OBE procedure can simultaneously realize decompression and fusion and is effective in the treatment of lumbar diseases.

## 1 Introduction

Lumbar degenerative disease is a prevalent condition in the elderly population, characterized by chronic low back pain that significantly reduces patients' quality of life (1). Lumbar spondylolisthesis, notable for its complex impact on spinal stability and neurological function, necessitates treatment approaches such as decompression, reduction, and interbody fusion (2, 3). Lumbar interbody fusion has been widely used in the treatment of lumbar degenerative diseases. Many modified interbody fusion techniques have been reported and used in lumbar diseases. The effective treatment of lumbar spondylolisthesis relies on the precise execution of pedicle screw fixation and interbody fusion, highlighting the significant technical demands of these procedures (4). This inherent complexity has driven advancements in surgical techniques and their clinical applications (5). Posterior lumbar interbody fusion (PLIF), a widely used interbody fusion method, is particularly effective for addressing a range of spinal disorders. This approach integrates adequate neural decompression procedures, including laminectomy, resection of the inferior articular processes, and removal of hyperplastic tissues, to ensure the safety and efficacy of the surgery. Although these techniques substantially improve outcomes in lumbar spondylolisthesis treatment, they are associated with challenges, such as significant surgical trauma and postoperative discomfort (6, 7).

Over the past decade, endoscopic spine surgery has made significant progress and has been widely utilized for treating disc herniation and spinal stenosis, particularly in lumbar decompression and discectomy procedures (8, 9). However, its application in lumbar interbody fusion remains in the early stages of development. At present, endoscopic interbody fusion is typically combined with percutaneous pedicle screw fixation (10, 11). Nonetheless, this approach has notable limitations: additional incisions increase tissue damage and postoperative pain, and the pre-insertion of interbody fusion cages hinders reduction. Despite these challenges, both decompression and fusion are essential components of endoscopic treatment for many lumbar conditions. Minimally invasive techniques that integrate decompression, reduction, and interbody fusion remain the ultimate objective for numerous surgeons.

Endoscopy provides a clear visualization of anatomical structures and minimizes blood loss by utilizing continual irrigation and applying water pressure to the tissues, making it the least invasive procedure. However, its drawbacks include relatively low surgical efficiency and a limited range of indications, primarily suitable for simple conditions. When addressing complex procedures such as lumbar spondylolisthesis, technical and equipment limitations make it challenging to achieve optimal therapeutic outcomes (12). Can we combine the conventional PLIF with the endoscopy to minimize the invasion and to realize adequate bilateral decompression, reduction, and interbody fusion? Thus, we introduce the endoscopic system to overcome the obstacles presented in minimizing PLIF. Herein, we present the endoscopy innovation of the one-hole bi-medium endoscopy (OBE) technique in the treatment of lumbar degenerative diseases.

## 2 Materials and methods

This is an innovative spinal endoscopic technique that incorporates a modified posterior lumbar interbody fusion, allowing for discectomy, bilateral decompression, endplate preparation, and cage and pedicle screw insertion through a single minimal incision with the assistance of an endoscopic system.

The surgical indications for this technique include (1) single-level lumbar disc herniation refractory to ≥3 months of conservative treatment; (2) central canal or foraminal stenosis with neurogenic claudication or radicular symptoms; (3) degenerative spondylolisthesis (Grades I–II) with radiographic and clinical evidence of instability; and (4) intervertebral disc space collapse with corresponding clinical and radiological findings. The contraindications include (1) lumbar spondylolisthesis (Grade II); (2) severe osteoporosis; (3) significant spinal scoliosis or rotational deformity (e.g., Cobb angle >20°); (4) active systemic or local infection at the surgical site; and (5) severe comorbidities precluding general anesthesia or prone positioning.

To more clearly demonstrate the key techniques and procedural standards during the surgery, we present a representative case and provide a detailed description of each specific step, along with critical intraoperative considerations. Through the analysis of this case, we aim to offer practical guidance for clinical implementation and help improve surgical safety in similar scenarios.

**Figure 1:**
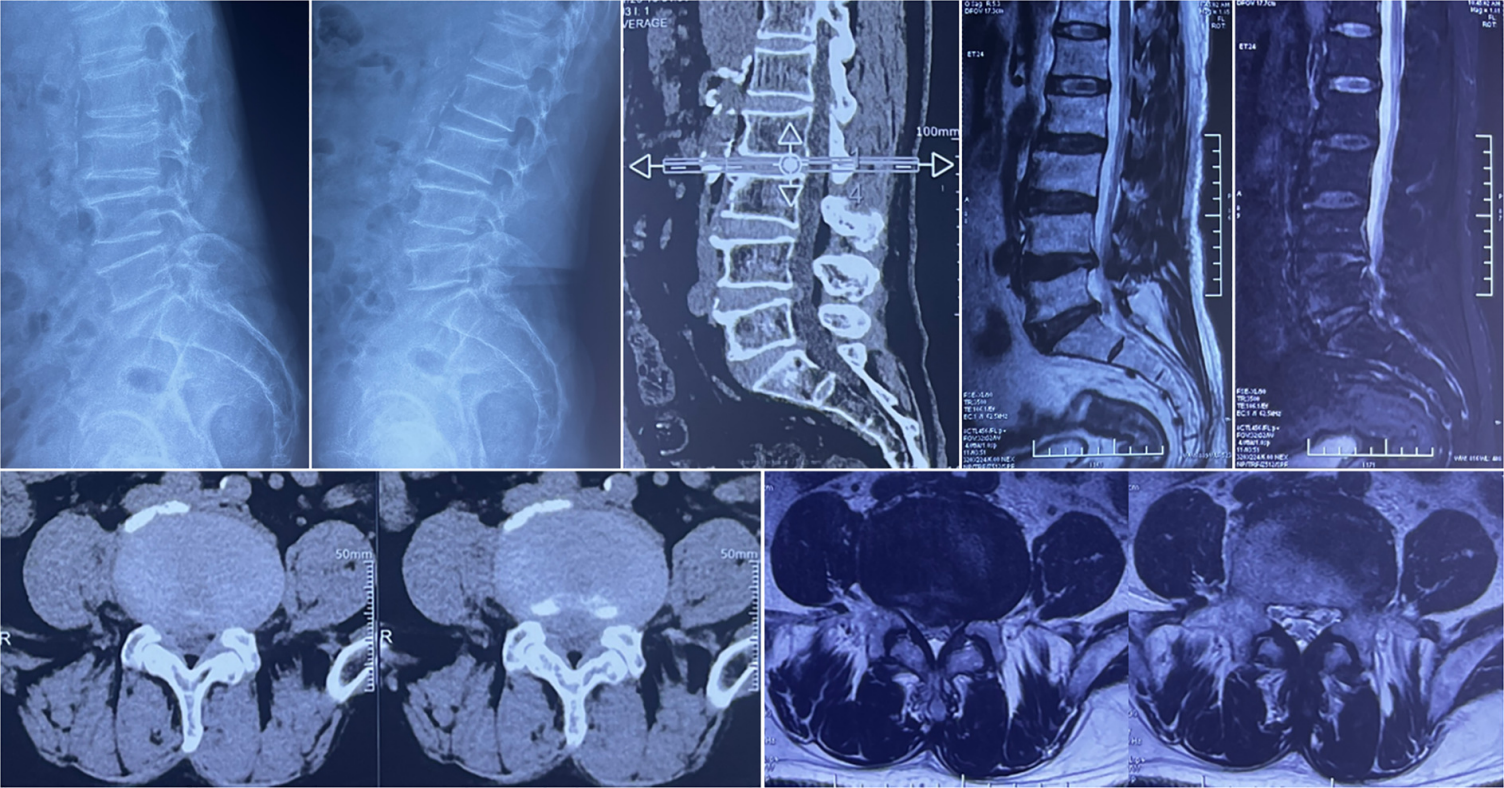
Preoperative imaging evaluations. Dynamic radiographs of the lumbar spine demonstrate instability at the L4–5 segment. CT and MRI scans reveal a herniated disc at the L4–5 level, accompanied by spinal canal stenosis.

A 68-year-old man presented with a 10-year history of severe backache, accompanied by both-sided radiculopathy and intermittent claudication for 3 years, which was initially treated with conservative treatment for 6 weeks. Tenderness pain was localized at the L4–5 level. Preoperative visual analog scale (VAS) scores for back and leg pain were 6 and 4, respectively. The Oswestry disability index (ODI) score was 17% preoperatively. The DR images in the flexion–extension position of the lumbar spine revealed instability at the L4/5 segment, while the CT reconstructed images showed spinal canal stenosis at the same level. Preoperative MRI scan showed degenerative disc disease and spinal canal stenosis at L4–5 (Figure 1).

Following induction of general anesthesia, the patient was positioned prone on a radiolucent table. Nerve root monitoring was performed during the procedure to prevent unexpected nerve injury. Cefazolin sodium (1 g) was intravenously injected 30 min before the incision.

**Figure 2:**
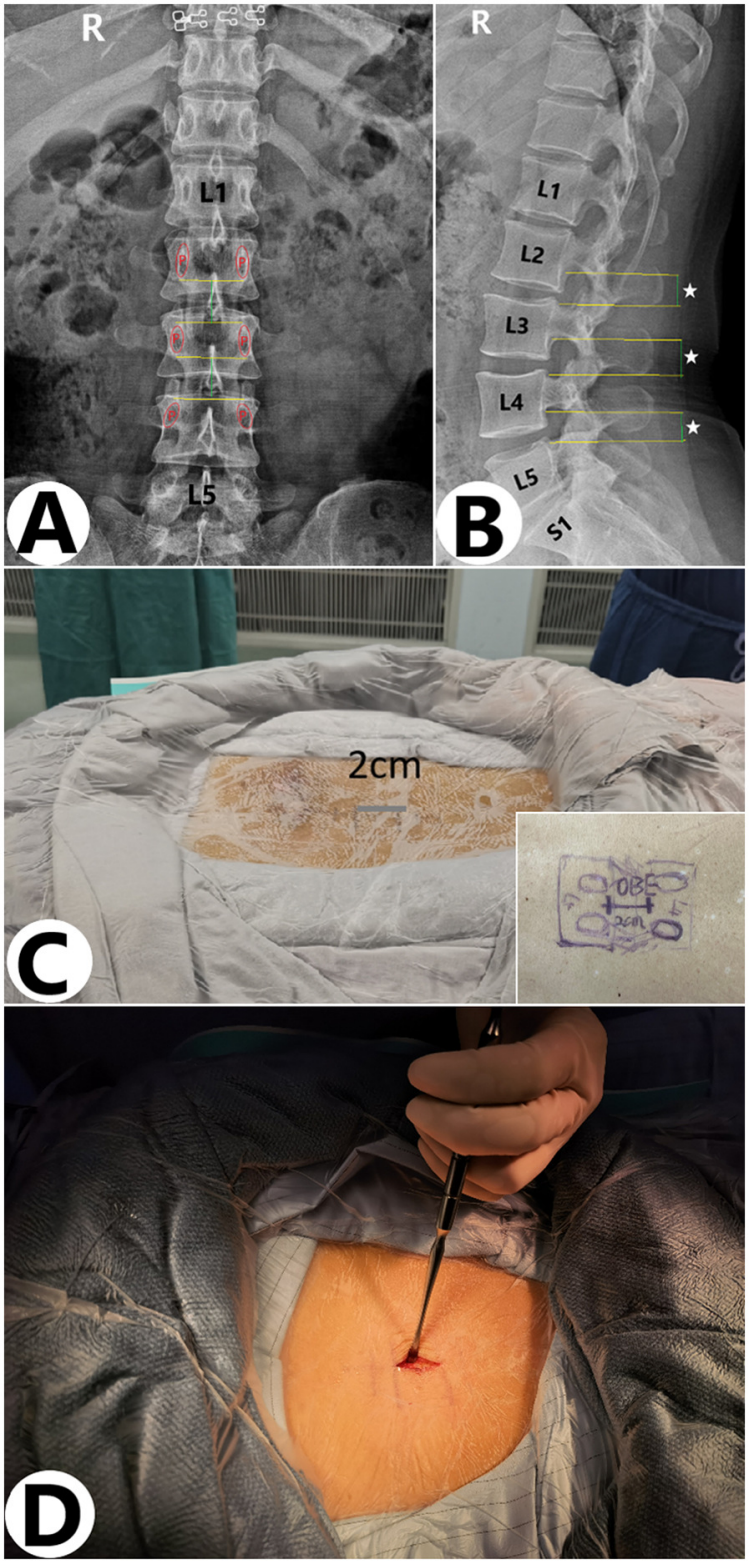
Surgical incision under x-ray guidance. (**A,B**) Under x-ray guidance, the incision extends from the lower edge of the superior vertebral arch to the upper edge of the inferior vertebral arch, forming a 2–3 cm posterior midline incision. (**C,D**) Actual size of the incision during surgery.

A 2 cm posterior midline incision was initially made to maintain stable hydrostatic pressure, thereby reducing bleeding and ensuring a clear endoscopic view. The paravertebral muscles were separated from the spinous process, and the lamina and articular process were exposed under direct visualization. A specialized distractor was used to further separate the paravertebral muscles, thus exposing the working zone. C-arm imaging was performed again to confirm the surgical segment (Figure 2). The tip of the customized retractor (Figure 5A) was placed against the base of the superior articular facet of the inferior vertebra, which stabilizes the working cannula and expands the operative field, thereby enhancing both direct and endoscopic visualization throughout the surgical procedure, without interfering with the subsequent resection of the superior articular process (SAP). The enlarged working passage enables the coexistence of the optical system, distractor, and working tools. Meanwhile, conventional tools used in open spinal surgery can also be used in the present technique, increasing the efficiency and shortening the operation duration. Most operative procedures are similar to those in traditional open spinal surgery, making this minimally invasive operation easier for surgeons to master.

**Figure 3:**
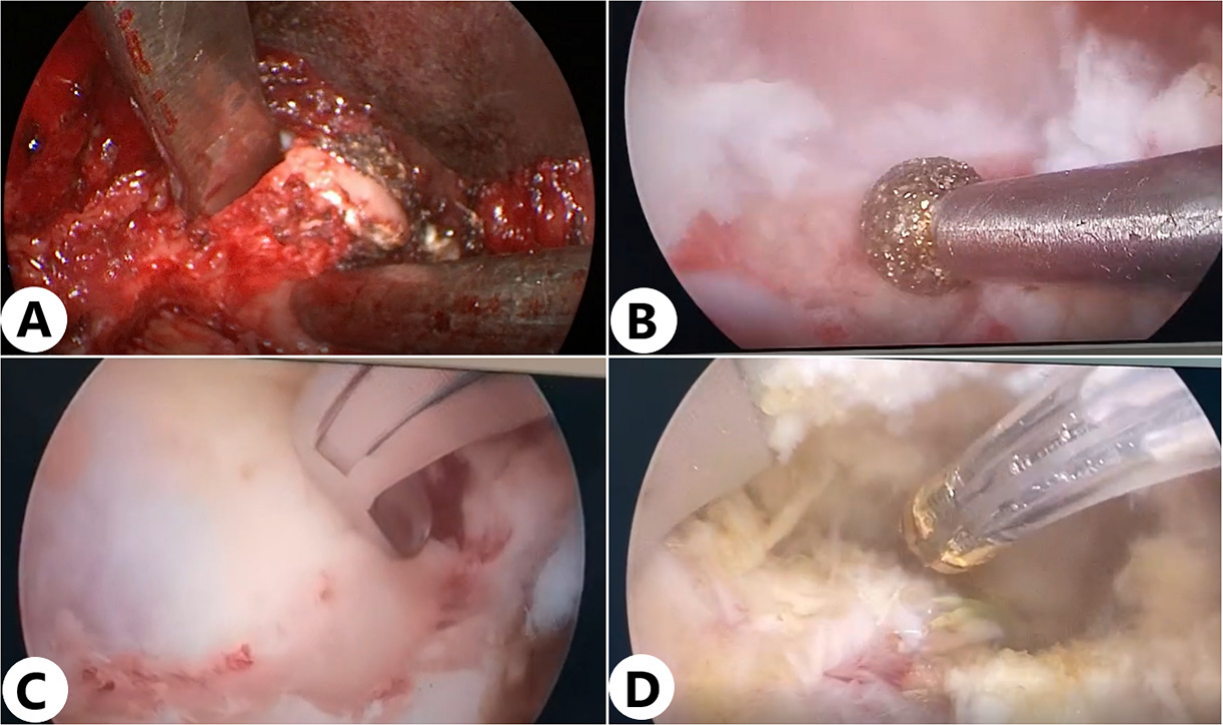
Intraoperative spinal canal decompression in the water medium. (**A**) Cutting the superior articular process with a bone cutter in an air medium. (**B,C**) In a water medium, the upper vertebral lamina is cut using a high-speed burr, while a Kerrison rongeur is used to remove the ligamentum flavum. (**D**) Ablation of tissue covering the dural sac with a radiofrequency probe.

A 4 mm outer diameter with a 0-degree angulated lens endoscope was introduced through the incision toward the facet joint without continuous saline irrigation. An osteotome was used to resect the inferior articular process (Figure 3A). Under endoscopic visualization with continuous saline irrigation, a high-speed drill (NSK Primado 2, Nakanishi Inc, Surgical Division, Kanuma, Japan) fitted with a 3 mm diamond burr was used to trim the medial part of the superior articular process and the cranial quarter of the inferior lamina until the bone became semitransparent (Figure 3B). Next, a 1 mm Kerrison punch was introduced to remove the interior thin layers, followed by 2 mm and 3 mm Kerrison punches to resect the ligamentum flavum until the exiting and traversing nerve roots were fully exposed (Figure 3C). Finally, a Smith & Nephew PLC (London, UK) radiofrequency probe with a 3.75 mm shaft was applied to ablate and coagulate the tissue overlying the dural sac (Figure 3D).

For the foraminal region, the endoscopic ultrasonic osteotome was used to remove the tips of the superior articular process close to the pedicle. Then a nerve hook was introduced to confirm no nerve compression; if necessary, the ligamentous and fibrous structures both within and outside the foramen were excised to ensure no compression on the exiting nerve root. Under continuous irrigation, this endoscopic decompression sequence parallels the steps performed under direct vision in conventional posterior lumbar interbody fusion.

**Figure 4:**
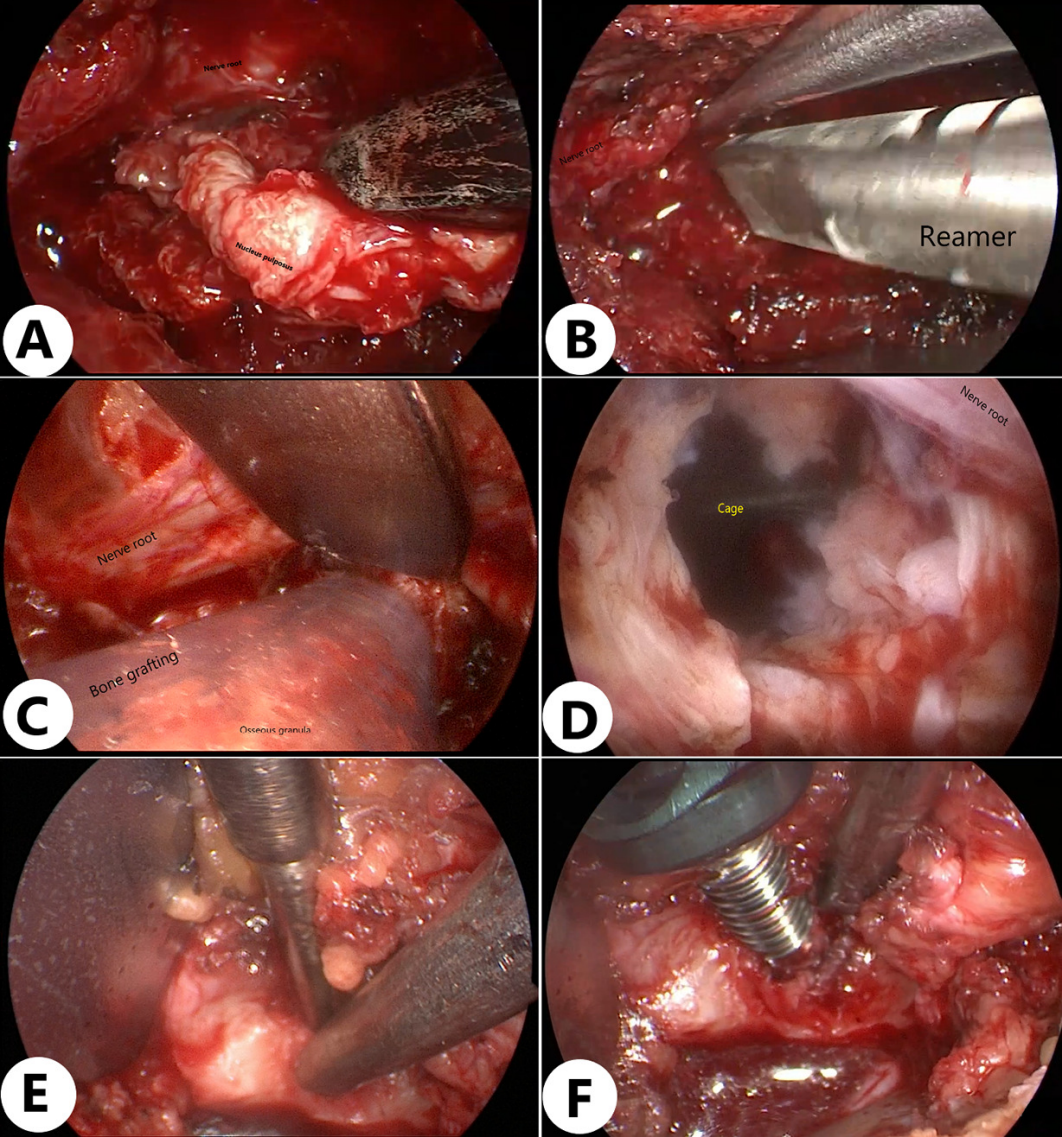
Key steps of interbody fusion in the air medium. (**A**) Thorough decompression was performed on the bilateral lateral recesses and intervertebral foramina, with effective removal of bone and soft tissue. After decompression, the neural structures could be clearly observed. (**B**) Under direct endoscopic visualization, the cartilage was removed using a curette and rasp to prepare the endplate while preserving its integrity. (**C,D**) After filling the intervertebral space with an allograft bone tray, an elliptical fusion device filled with bone material is precisely inserted into the intervertebral space under endoscopic direct vision. The entire insertion process is performed under direct visualization, effectively avoiding damage to surrounding nerves and ensuring the accurate placement of the fusion device. (**E,F**) After clearly identifying the entry point for the pedicle screw under endoscopic guidance, the screw is accurately directed into its predetermined position.

The intervertebral disc was resected after decompression. The ventral epidural vessels overlying the disc were coagulated using the RF probe or bipolar coagulation. The nucleus pulposus was removed by a scalpel and pituitary rongeurs of variable sizes (Figure 4A). Under direct endoscopic visualization, the endplates were prepared using curettes and rasps to remove the cartilage while preserving the integrity of the endplates (Figure 4B). Liquid irrigation improves hemostasis and visualization, allowing surgeons to easily distinguish ligament, nerve, and disc under clear and magnified visualization. Endoscopic visualization under an air medium can significantly improve the efficiency and safety. Meanwhile, bi-medium, e.g., air medium and liquid irrigation, can be easily adapted according to different procedures and preferences.

Allograft bone was delivered into the intervertebral space in an air medium. A bullet-shaped cage filled with bone was inserted into the intervertebral space under direct endoscopic visualization, preventing neural damage (Figures 4C,D). The endoscope was used for direct observation of cage insertion.

After decompression, the medial wall of the pedicle was identified. Then the entry point of the pedicle screw was identified. Endoscopic visualization in an air medium clearly displayed many structures, facilitating the efficient use of osteotomes and other instruments. The pedicle screw reached the entry point through the existing incision under endoscopic visualization (Figures 4E,F). The fusion was assisted by bilateral rods.

## 3 Results

**Figure 5:**
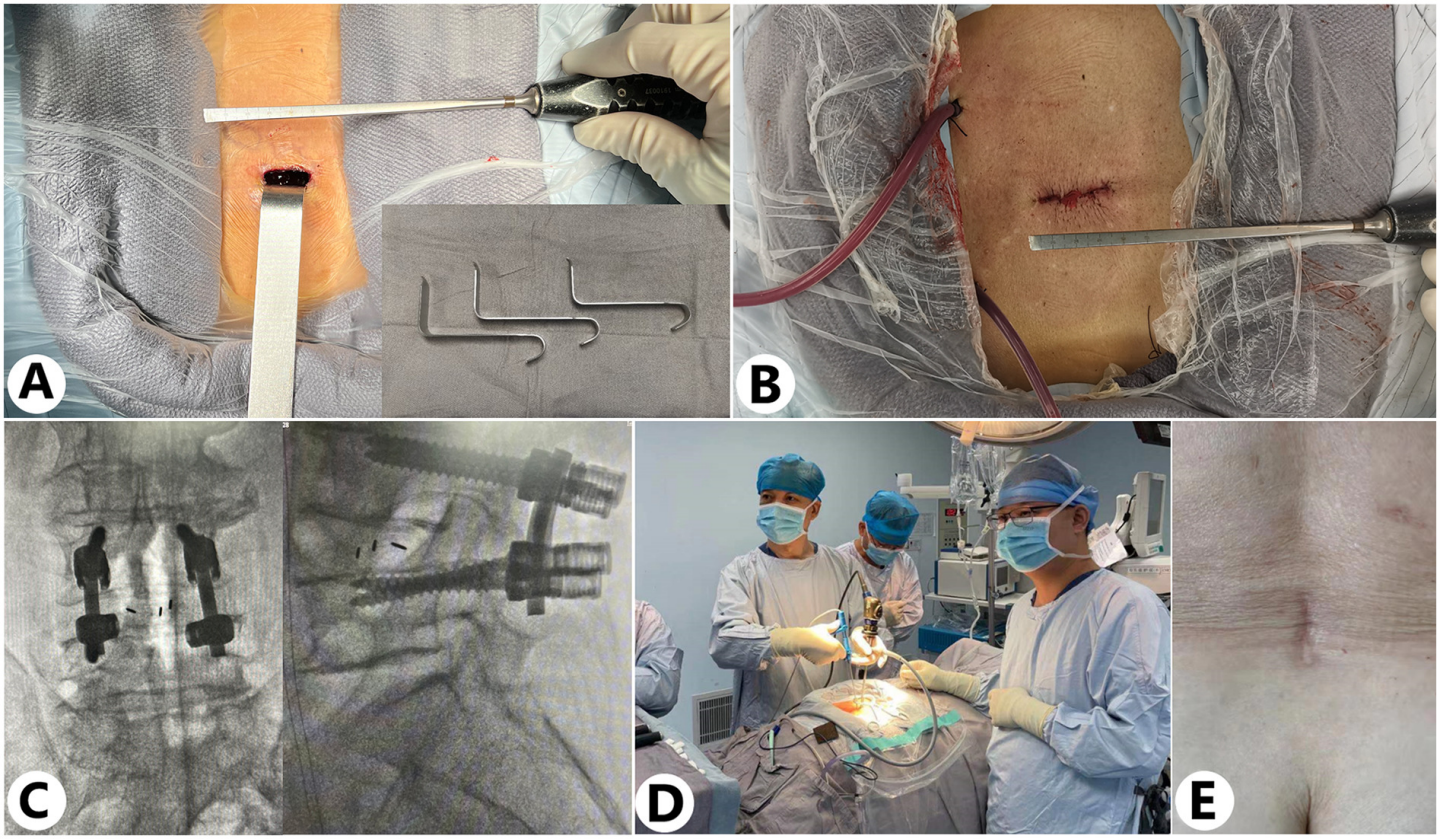
Intraoperative and postoperative imaging evaluations and surgical outcomes. (**A,B**) Incision size at the beginning and end of the surgery [the customized retractor is shown in the lower right corner of (**A**)]. (**C**) Intraoperative x-ray imaging was used to monitor the position of the screw-rod construct. (**D**) Intraoperatively, the OBE technique imaging system is used for real-time visual monitoring of manipulations in the surgical area. (**E**) Postoperative wound healing status.

The operation time was 176 min, with an estimated blood loss of 110 mL. No surgical complications occurred in this patient. During the surgery, x-ray clearly confirmed the correct position of the internal fixation device. After suturing, the incision measured approximately 3 cm (Figure 5). Postoperatively, the VAS leg score was reduced to 3 from a preoperative score of 7, and the VAS back score was reduced to 2 from a preoperative score of 4. The ODI score improved from 75% before surgery to 19% at the last follow-up.

**Figure 6:**
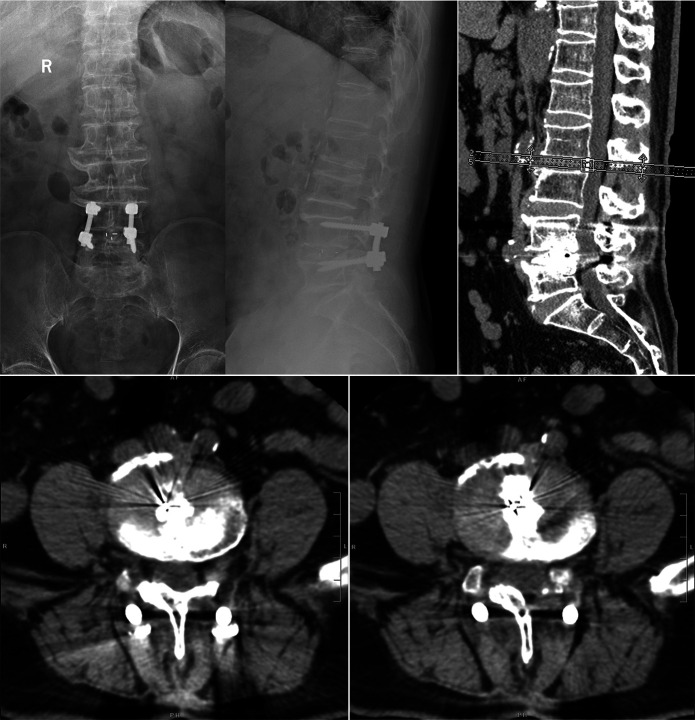
Three-year postoperative follow-up x-ray and CT imaging. At the 3-year postoperative follow-up, radiographic and CT examinations revealed the formation of continuous bony bridges in the grafted region, with no evidence of bony gaps or pseudarthrosis, confirming successful bone fusion. The internal fixation devices, including screws, rods, and interbody fusion cages, were intact and maintained in their normal positions, with no signs of displacement, loosening, or breakage.

At the 3-year postoperative follow-up, radiographic and CT images were jointly evaluated by radiologists and spinal surgeons to assess the patient's condition. Imaging studies revealed continuous formation of bony bridges in the grafted region, with no evidence of bony gaps or pseudarthrosis, indicating successful bone fusion. The internal fixation devices, including screws, rods, and interbody fusion cages, remained in normal positions, with no signs of displacement, loosening, or breakage. In addition, the corrected spinal physiological curvature, particularly lumbar lordosis, was well-maintained. CT further confirmed that the spinal canal volume was normal, with no significant narrowing or abnormalities observed, and no evidence of nerve compression (Figure 6). Overall, the follow-up findings indicated favorable postoperative recovery, with imaging results supporting the achievement of surgical objectives.

## 4 Discussion

Degenerative spondylolisthesis is often accompanied by spinal canal, lateral recess, and foraminal stenosis, which necessitate adequate decompression and reduction. Thus, bilateral decompression of the lateral recess and foramina is necessary (13). It is difficult to realize complete decompression using unilateral biportal endoscopy (UBE) in the treatment of lumbar diseases. Drawing from endoscopic lumbar interbody fusion and modified PLIF techniques, we developed the one-hole bi-medium endoscopy lumbar interbody fusion (OBE-LIF) technique. The enlarged working channel enables efficient and complete decompression of the spinal canal, lateral recess, and foramina. Adequate decompression can minimize the risk of nerve root injury after reduction. Meanwhile, the use of tools in open surgery can realize the complete reduction in this minimally invasive operation. The simplified endplate decortication under endoscopic visualization may help improve fusion quality and potentially reduce the risk of cage subsidence. Moreover, efficient intraoperative manipulation can contribute to reduced blood loss and shorter surgical time.

The OBE technique of our minimally invasive lumbar interbody fusion under endoscopic visualization originates from the combination of the endoscopic system and conventional PLIF. Because of the introduction of the endoscopic system, the OBE-LIF technique offers several advantages over the conventional procedure, including clear visualization, minimal invasion, decreased blood loss, enhanced recovery after operation, and shortened hospital stays.

Biportal endoscopic spine surgery (BESS) has been widely used in the treatment of spinal stenosis (14, 15). It is a viable alternative to the microscopic technique for lumbar canal stenosis decompression with similar operative time, clinical outcomes, and complications (16). However, most studies have reported the use of BESS without interbody fusion and internal fixation (17, 18). Thus, BESS has not been widely used in the treatment of spondylolisthesis. The OBE-LIF technique is capable of achieving bilateral decompression, pedicle screw fixation, and interbody fusion through a single incision, thereby extending its applicability beyond that of certain endoscopic procedures.

The midline incision in the OBE-LIF technique differs from that in the UBE operation. We made only one midline minimal incision which is longer than that in the UBE technique, but through which we can complete bilateral decompression, interbody fusion, and pedicle screw fixation. During the process of decompression, only a 3 cm length incision was made, which can accommodate the optical system, liquid irrigation, and most of the tools, such as the osteotome, pituitary rongeurs, Kerrison punches, high-speed drill, RF probe, various shavers, curettes, and cages. The OBE-LIF technique procedure allows pedicle screw insertion under direct endoscopic visualization, eliminating the need for continuous fluoroscopic guidance and distinguishing it from other endoscopic interbody fusion techniques that rely on fluoroscopy.

**Figure 7:**
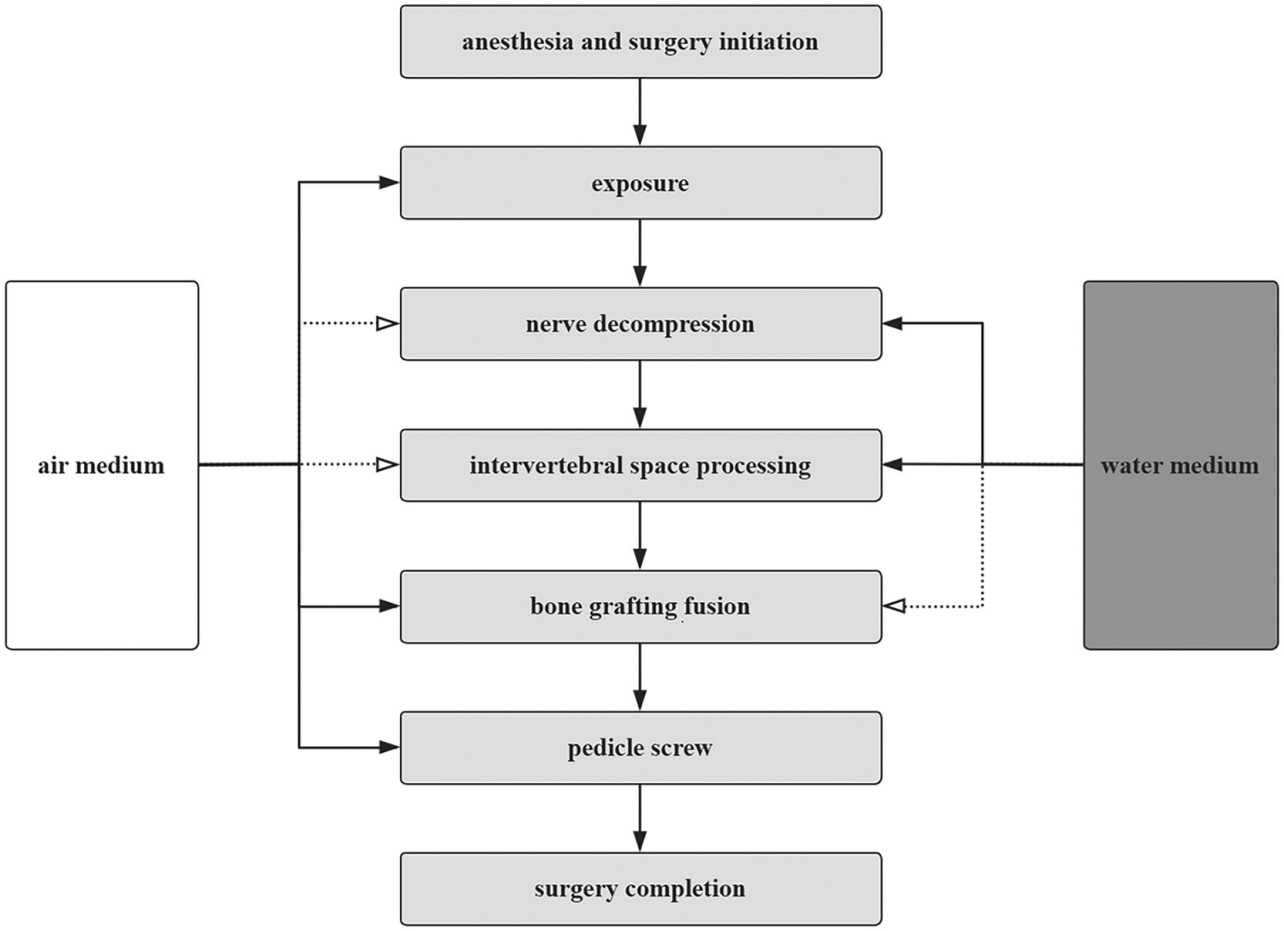
Flowchart. In the OBE technique, the selection of operating media for each step is indicated, with solid lines representing the primary operating medium and dashed lines representing the auxiliary operating medium.

Endoscopic visualization without continuous irrigation is advantageous during the use of an osteotome in removing the articular process and lamina (Figure 7). However, continuous irrigation can give clearer visualization in the process of decompression and in the usage of a high-speed drill. Surgeons experienced in endoscopic operations are familiar with endoscopic visualization and may experience a shorter learning curve in mastering the OBE-LIF technique. Although the OBE-LIF technique incorporates both non-irrigated and continuous-irrigation endoscopic views familiar to surgeons trained in uniportal or biportal techniques, mastery of the combined gas–liquid workflow still benefits from a structured training program. At our institution, the mean operative time decreased from 192.7 ± 12.3 min in the initial 15 cases to 145.4 ± 15.7 min after case 50. Therefore, with targeted hands-on training and a stepwise escalation in case complexity, the learning curve of the OBE-LIF technique is not steep and is overall comparable to, or even more favorable than, that of uniportal or biportal endoscopic lumbar interbody fusion techniques (19, 20).

In OBE-LIF procedures, pedicle screw insertion is performed after decompression. The entry point is identified under the endoscopic visualization. After finishing decompression, the exposed medial wall of the pedicle indicates the optimal trajectory of the pedicle screw. Based on the identified entry point and trajectory, the pedicle screw can be safely inserted without the guidance of fluoroscopy. Besides, cage insertion is also performed under direct visualization. Thus, the OBE-LIF technique does not necessarily require inserting a pedicle screw under continuous fluoroscopy, which is different from other endoscopic interbody fusion techniques based on fluoroscopic guidance (21, 22).

Nerve root injury is a common complication in endoscopic interbody fusion (23). Studies reported different rates of exiting nerve irritation or injury, which ranged from 0% to 22% in uniportal endoscopic TLIF (24). An exiting nerve root injury usually happens during the cage insertion through Kambin's triangle. To get enough space in the cage insertion, a significant portion of the SAP and the base of the spinous process were resected in our OBE-LIF technique. No irreversible nerve injury happened in our cases.

Spinal canal, lateral recess, and foraminal stenosis exist simultaneously in degenerative spondylolisthesis. Bilateral decompression of the lateral recess and foramina is necessary. Foraminal stenosis is common in degenerative spondylolisthesis. Thus, SAP resection is usually necessary, which is difficult to complete in endoscopic TLIF (24).

Since then, we have completed more than 100 operations using the OBE technique, covering diseases such as lumbar disc herniation, lumbar spinal stenosis, and lumbar spondylolisthesis. All procedures were performed by a dedicated team. The lead surgeon has spinal surgery experience over 30 years. All other assistants have more than 5 years of experience in spine surgery. Patients experienced satisfactory relief of symptoms post-surgery, and all procedures were successfully completed with an average duration of 159 min and average blood loss of 137 mL. The average hospital stay was 4.6 days. These data are shown in Table 1. Compared to preoperative levels, VAS scores significantly decreased at 3 days and 3 months post-surgery, while ODI scores decreased from 65% preoperatively to 26% at 3 months and 14% at the 2-year follow-up (Table 2). All patients underwent postoperative follow-up for a duration of 2–3 years. During this period, patients generally expressed high satisfaction with the surgical outcomes, with VAS scores recorded at ≤3, indicating sustained pain relief and favorable long-term results.

**Table 1:** Patient demographics and surgical characteristics.

Characteristic	*N* = 123^a^	95% CI^b^
Age	60 ± 8	55.6–68.4
BMI (body mass index)	25.5 ± 3.2	24.9–26.1
Surgery duration (min)	159 ± 23	154.4–162.8
Blood loss (mL)	137 ± 45	130.2–146.1
Incision length (cm)	3.15 ± 0.17	3.1–3.2
Postoperative hospital stay (days)	4.59 ± 1.61	4.3–4.9

^a^ Mean ± SD.

^b^ CI, confidence interval.

**Table 2:** Paired *t*-test comparison of pre-op and post-op VAS/ODI scores.

Characteristic	Pre-op (mean ± SD)	Time	Post-op (mean ± SD)	*t*	*p*
VAS.leg	7.0 ± 1.03	3 days	3.8 ± 0.70	29.25	<0.01
		3 months	2.5 ± 0.58	43.76	<0.01
		12 months	1.6 ± 0.57	50.41	<0.01
		2 years	1.4 ± 0.79	46.75	<0.01
VAS.back	6.0 ± 1.10	3 days	2.7 ± 0.81	24.48	<0.01
		3 months	2.0 ± 0.74	32.66	<0.01
		12 months	1.1 ± 0.51	43.23	<0.01
		2 years	0.8 ± 0.54	44.45	<0.01
ODI.pre	73.9 ± 10.48	3 days	39.0 ± 7.10	32.05	<0.01
		3 months	26.0 ± 6.02	44.35	<0.01
		12 months	16.5 ± 5.19	53.98	<0.01
		2 years	14.2 ± 6.92	53.32	<0.01

Regarding intraoperative and postoperative complications, one patient sustained a dural tear during surgery, while two others developed transient motor and sensory deficits in the lower limbs postoperatively. Another patient presented with a postoperative infection. All affected individuals received appropriate treatment and were successfully discharged. At the 6-month follow-up, six patients demonstrated unsatisfactory bone fusion, prompting the initiation of anti-osteoporosis therapy. By the 1-year follow-up, two patients continued to exhibit impaired bone healing, and one patient experienced breakage of the internal fixation. At 2 years postoperatively, adjacent segment spondylolisthesis was identified in one patient, which was managed with conservative treatment.

Surgeries that typically required a 10 cm incision could be completed through a 3 cm incision using the OBE technique, resulting in minimal surgical trauma, high safety, and high patient satisfaction. Although the overall incidence of postoperative complications with the OBE technique was not significantly different from that of traditional open surgery, the OBE technique demonstrated clear advantages in terms of reduced intraoperative and postoperative blood loss, shorter hospital stays, and better cosmetic satisfaction with the incision. Moreover, OBE surgery can be standardized, offering high procedural stability with minimal tissue disruption.

We acknowledge that the OBE technique is currently used in lumbar degenerative conditions. A narrow working channel helps to keep a stable fluid pressure, limiting its popularity in cases requiring wide exposure. Therefore, in patients with high-grade spondylolisthesis or severe rotational deformities, traditional open surgery may be more useful to complete decompression and reduction.

## 5 Conclusions

The OBE-LIF technique is a minimally invasive endoscopic posterior spinal fusion. Endoscopic visualization under either air or liquid medium can clearly present anatomical structures. Thorough decompression, interbody fusion, and posterior fixation are technically feasible and safe to nerve roots in the OBE-LIF technique.

## Data Availability

The raw data supporting the conclusions of this article will be made available by the authors, without undue reservation.
